# All tyrosine kinase inhibitor-resistant chronic myelogenous cells are highly sensitive to Ponatinib

**DOI:** 10.18632/oncotarget.692

**Published:** 2012-11-14

**Authors:** Ophélie Cassuto, Maeva Dufies, Arnaud Jacquel, Guillaume Robert, Clémence Ginet, Alix Dubois, Amine Hamouda, Alexandre Puissant, Fredéric Luciano, Jean-Michel Karsenti, Laurence Legros, Jill Patrice Cassuto, Pascal Lenain, Patrick Auberger

**Affiliations:** ^1^ C3M/ INSERM U1065 Team Cell Death, Differentiation, Inflammation and Cancer, Nice, France; ^2^ Equipe Labellisée Ligue Nationale contre le Cancer 2011-2013, Paris, France; ^3^ Service d'Hématologie Clinique et de Transplantation, CHU de Nice, France; ^4^ Centre Henri Becquerel, CLCC, Rouen, France

**Keywords:** CML, BCR ABL, TKI, Resistance, Ponatinib

## Abstract

The advent of tyrosine kinase inhibitor (TKI) therapy has considerably improved the survival of patients suffering chronic myelogenous leukemia (CML). Indeed, inhibition of BCR-ABL by imatinib, dasatinib or nilotinib triggers durable responses in most patients suffering from this disease. Moreover, resistance to imatinib due to kinase domain mutations can be generally circumvented using dasatinib or nilotinib, but the multi-resistant T315I mutation that is insensitive to these TKIs, remains to date a major clinical problem. In this line, ponatinib (AP24534) has emerged as a promising therapeutic option in patients with all kinds of BCR-ABL mutations, especially the T315I one. However and surprisingly, the effect of ponatinib has not been extensively studied on imatinib-resistant CML cell lines. Therefore, in the present study, we used several CML cell lines with different mechanisms of resistance to TKI to evaluate the effect of ponatinib on cell viability, apoptosis and signaling. Our results show that ponatinib is highly effective on both sensitive and resistant CML cell lines, whatever the mode of resistance and also on BaF3 murine B cells carrying native BCR-ABL or T315I mutation. We conclude that ponatinib could be effectively used for all types of TKI-resistant patients.

## INTRODUCTION

Chronic myelogenous leukemia (CML) is a disease of the hematopoietic stem cell, characterized by the t(9 ; 22) q(34 ; q11) translocation encoding the oncoprotein BCR-ABL [1]. Patients suffering CML benefit from new-targeted therapies based on the use of tyrosine kinase inhibitors (TKIs). Three TKIs, imatinib, dasatinib and nilotinib that target BCR-ABL are routinely given with success as first or second-line treatment for this disease, but resistance occurs in a significant proportion of CML patients [2-4]. Several BCR-ABL-dependent mechanisms of resistance to imatinib have been identified, such as BCR-ABL point mutations involving or not the kinase domain or increased expression of BCR-ABL [5]. In addition to these BCR-ABL dependent events, several BCR-ABL independent mechanisms have been also reported, including increased expression and/or activation of tyrosine kinases including, the Src tyrosine kinases Lyn [6, 7] and Fyn [8, 9] and the receptor tyrosine kinase Axl [10, 11] as noticeable examples.

Regarding BCR-ABL kinase mutations, the multi-resistant T315I one remains a crucial clinical challenge, since until recently, there is no effective treatment for the patients carrying this particular type of mutation. Ponatinib is a multi-targeted kinase inhibitor that exhibits high activity against the T315I mutation but also other BCR-ABL kinase mutants *in vitro* [12, 13]. In addition, ponatinib has proven efficacy in mouse models of CML and was also found to be effective in a small cohort of patients with T315I mutations in two recent clinical trials [12, 14, 15] (phase 1: NCT00660920 ; phase 2: NCT01207440; http://www.clinicaltrials.gov).

Surprisingly, although ponatinib represents a promising molecule for patients with BCR-ABL mutations, its mechanism of action has not been extensively studied, more particularly in imatinib-resistant CML cells with no BCR-ABL mutation. In this line, we have previously generated several imatinib-resistant cell lines from the parental K562, JURLMK1 and Lama CML cell lines [8, 16, 17].

In the present study, we took advantage of the availability of these cell lines to evaluate the effect of ponatinib in comparison to other TKIs on cell metabolism, proliferation and apoptosis. In addition, we used the murine BaF3 cell line carrying either a wild-type BCR-ABL protein or its T315I and G250E mutated counterparts to decipher the mechanisms of action of this TKI. We show that ponatinib is highly efficient to induce cell growth inhibition and induction of apoptosis on different imatinib CML cell lines, whatever their mode of resistance.

## RESULTS

**Figure 1:**
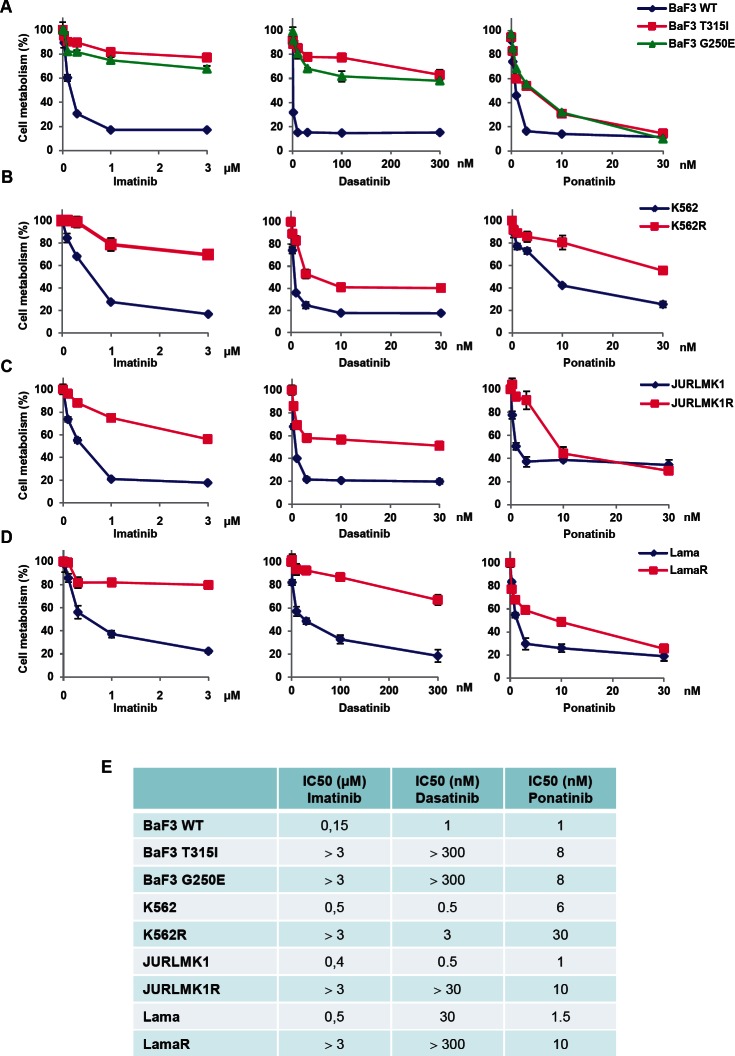
Ponatinib induces a loss of viability in different TKI-resistant cell lines (A to D) The BaF3 cell line (A) and the K562 (B), JURLMK1 (C) and Lama (D) CML cell lines were incubated for 48h at 37°C with increasing concentrations of imatinib (left panel), dasatinib (middle panel) or ponatinib (right panel) or left untreated and cell metabolism was measured using the XTT assay as described in the materials and methods section. (E) IC50 values for each TKI are given for each cell lines.

The effect of ponatinib on the viability of several imatinib-resistant cell lines was evaluated in comparison with that of imatinib and dasatinib. Cell lines were incubated for 48h with increasing concentrations of imatinib, dasatinib or ponatinib and cell viability was assessed using the XTT assay. We first investigated the effect of these ITKs on the viability of murine Ba/F3 cells, carrying wild type (WT) BCR-ABL, T315I or G250E-BCR-ABL mutation. As shown in Figure 1A, BaF3 cells expressing native BCR-ABL were highly sensitive to imatinib, dasatinib and ponatinib, whereas BaF3-T315I-BCR-ABL and BaF3-G250E-BCR-ABL cells were resistant to both imatinib and dasatinib. By contrast, ponatinib induced loss of cell viability in BaF3 cells carrying the T315I or G250E mutation, in agreement with previous results from the literature [12, 18]. As expected, imatinib-resistant K562, Lama and JURLMK1 cell lines were resistant to high doses of imatinib and were also cross-resistant to dasatinib (Figure 1B, C and D). However, the three resistant cell lines exhibited high sensitivity to ponatinib, even though a higher concentration of ponatinib was necessary to achieve an equivalent loss of cell viability in sensitive versus resistant CML cells. Globally, high doses of ponatinib (30nM) efficiently killed all imatinib-resistant cell lines.

The IC50 values for the ponatinib effect's in parental CML cells and for the different imatinib-resistant cells lines were very close (1 to 6nM versus 8 to 30nM) (Figure 1E). The IC50 values for imatinib were 0,15 to 0,5μM in parental cells versus more than 3μM in the resistant one. In addition, the IC50 values for dasatinib were 0.5 to 30nM in parental cells and 30 to more than 300nM in resistant cells. As a whole our findings confirmed the efficacy of ponatinib on the T315I mutation, but also highlighted the notion that ponatinib is effective on all types of imatinib-resistant CML cells, whatever their mode of resistance. Importantly, the IC50 values obtained for ponatinib in the present study for BaF3-WT-BCR-ABL, BaF3-T315I-BCR-ABL and BaF3-G250E-BCR-ABL (1nM, 8nM and 8nM respectively) were very similar to the one reported previously by O’ Hare et al. (0.5, 11 and 4.1nM respectively) [12].

Moreover, the IC50 values for parental K562 CML cells (6nM) and Lama CML cells (1nM) were also in good agreement with the one previously reported in the literature (3.9 and 0.5nM, respectively) [12].

### Ponatinib induces cell death in different TKI cell lines

**Figure 2:**
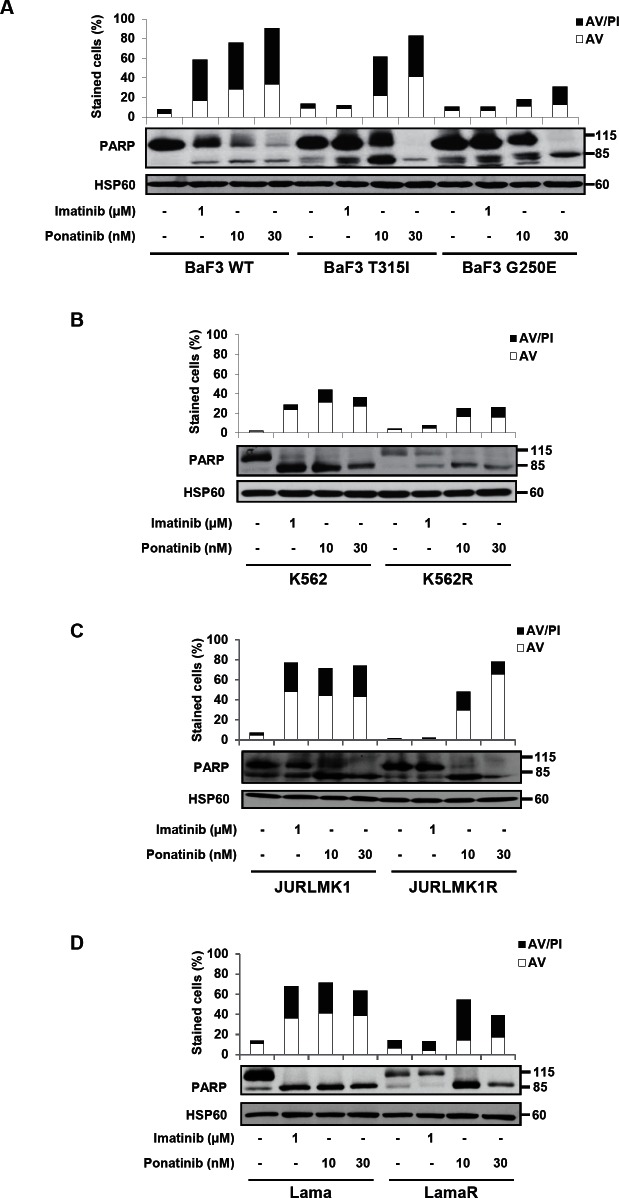
Ponatinib induces cell death in different TKI-resistant cell lines The BaF3 cell line (A) and the K562 (B), JURLMK1 (C) and Lama (D) CML cell lines were incubated for 48h at 37°C with either imatinib (1μM) or ponatinib (10 or 30nM). Cells were then stained with the PI/ annexin-V-fluos staining kit according to the manufacturer's indications. Histograms show both annexin-V^+^/PI^−^ cells (open bars) and annexin-V^+^/PI^+^ cells (filled bars). Cleavage of PARP was analyzed by western blot in each cell line.

It is well established that TKIs induced different types of cell death, including apoptosis in CML cell lines [8, 9, 11, 16, 19, 20]. Therefore, we next used Annexin V and PI staining to assess cell death in Ba/F3 cells carrying wild-type or mutated BCR-ABL proteins and in CML cell lines treated with either a maximal dose of imatinib or increasing concentrations of ponatinib. Ponatinib was found to trigger apoptosis in both BaF3-WT and BaF3-T315I cells and to a less extend in BaF3-G250E cells (Figure 2A). Apoptosis induction by ponatinib was confirmed by the cleavage of poly-ADP-ribose polymerase (PARP), a caspase 3 substrate. Indeed, cleavage of PARP in its 85kDa fragment was detected in the three BaF3 cell lines (Figure 2A). Imatinib (1uM) was shown to significantly increased the number of Annexin V positive cells, a hallmark of apoptosis, in the K562, JURLMK1 and Lama CML cell lines, but not in their imatinib-resistant counterparts (Figure 2B, C and D). Ponatinib (10-30nM) was as efficient as 1uM imatinib to trigger apoptosis in the three sensitive cell lines. Conversely to imatinib, ponatinib was found to induce apoptosis in all three imatinib-resistant cells lines, even though sensitivity to ponatinib was lower in K562R and LamaR cells as compared to JURLMK1R cells. Apoptosis induction by ponatinib was confirmed by PARP cleavage in each sensitive and resistant cell lines (Figure 2B, C and D).

### Ponatinib inhibits the clonogenic potential of different TKI-resistant cell lines

To investigate the effect of ponatinib on CML cell growth, we next performed clonogenic assays using the different Imatinib-sensitive and resistant cell lines described above. BaF3 cells carrying WT BCR-ABL protein failed to form colonies in soft agar in the presence of imatinib (Sup Figure 1A, blue histogram).

By contrast, BaF3 cells carrying the T315I or G250E-BCR-ABL mutation were fully resistant to imatinib treatment (Sup Figure 1A, red and green histograms). Importantly, ponatinib efficiently impaired colony formation in WT, T315I and G250E-BaF3 cell lines with a maximal effect at 10nM (Sup Figure 1A).

In addition, as illustrated on Sup Figure 1B, C and D, the clonogenic potential of all imatinib-sensitive cell lines was abolished in the presence of low doses of imatinib (0.3 to 1μM). As expected, all imatinib-resistant cells lines maintained high clonogenic potential in the presence this drug. Importantly, ponatinib induced a dose-dependent decrease of the clonogenic potential of all three imatinib-resistant clones. Maximal inhibition was achieved for 10 or 30nM of ponatinib depending on the cell line (Sup Figure 1A, B and C), JURLMK1 cells being highly sensitive to the effect of the drug (Sup Figure 1D). From the data described in Figures 1, 2 and in Sup Figure 1, we conclude that ponatinib exerts its anti-leukemic effect in different imatinib-sensitive and resistant CML cell lines through both inhibition of their proliferative potential and induction of apoptosis.

### Ponatinib differently affects cell signaling in TKI-resistant cell lines

**Figure 3:**
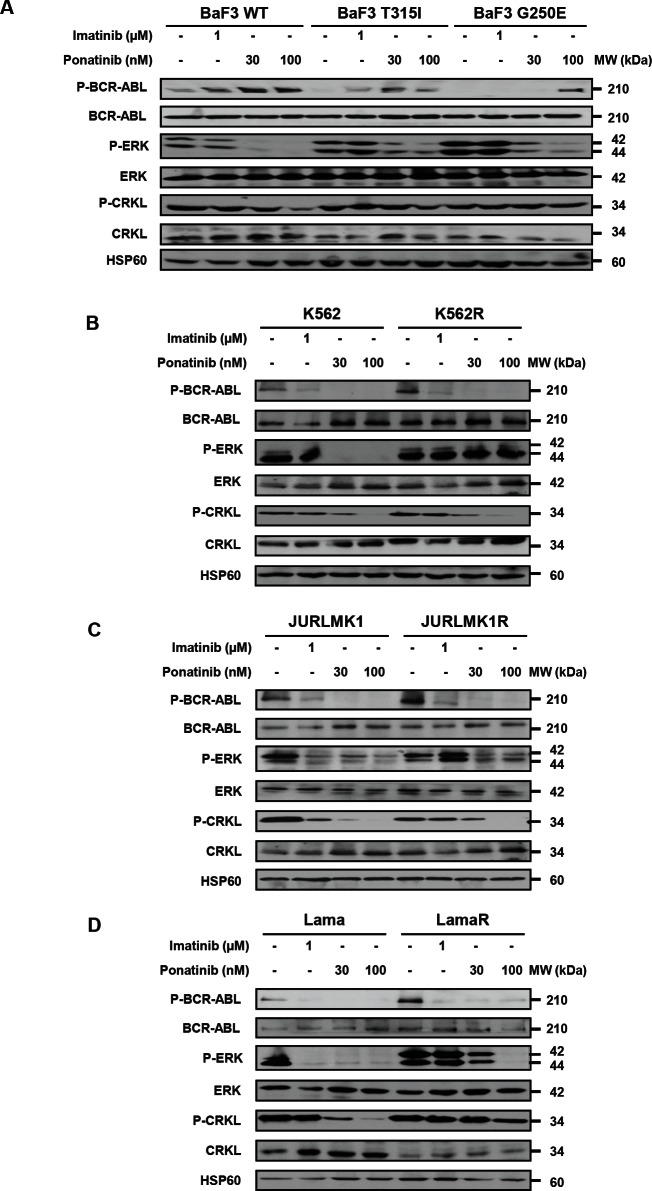
Ponatinib differently affects cell signaling in TKI-resistant cell lines The BaF3 cell line (A) and the K562 (B), JURLMK1 (C) and Lama (D) CML cell lines were incubated for 4h at 37°C with either imatinib (1μM) or ponatinib (30 or 100nM). BCR-ABL, ERK and CRKL phosphorylation status and expression were analyzed by Western blotting.

We next analyzed the effect of imatinib and ponatinib on signal transduction in the three BaF3 cell lines and in the different imatinib-resistant CML cell lines. To this end, we performed western blot analysis of several direct or indirect substrates of BCR-ABL. In BaF3-WT-BCR-ABL and T315I cells, ponatinib efficiently inhibited BCR-ABL phosphorylation, but dephosphorylation of CRLK necessitated higher doses of ponatinib (Figure 3A). Surprisingly, the effect of ponatinib was less pronounced on BaF3-G250E-BCR-ABL cells. As expected, Imatinib failed to inhibit BCR-ABL and CRKL phosphorylation in BaF3 cells carrying mutated BCR-ABL.

We have previously reported that K562R cells exhibited constitutive activation of ERK1/2 as a mechanism of resistance to this TKI [9, 17]. Regarding the two other resistant cell lines (LamaR and JURLMK1R) the mechanism of resistance to imatinib is not fully understood but the data reported here in clearly show a constitutive activation of ERK1/2 also in these cells [9, 11]. Nevertheless ponatinib inhibited BCR-ABL phosphorylation and, to a less extent CRKL phosphorylation (Figure 3B, C and D). Finally, maintenance of ERK1/2 activation was detected in JULMK1R and LamaR CML cells treated with ponatinib as it is the case in K562R cells (Figure 3B and [9]).

### Ponatinib is highly efficient on CD34+ cells but not on PBMC from a CML patient at diagnosis

**Figure 4:**
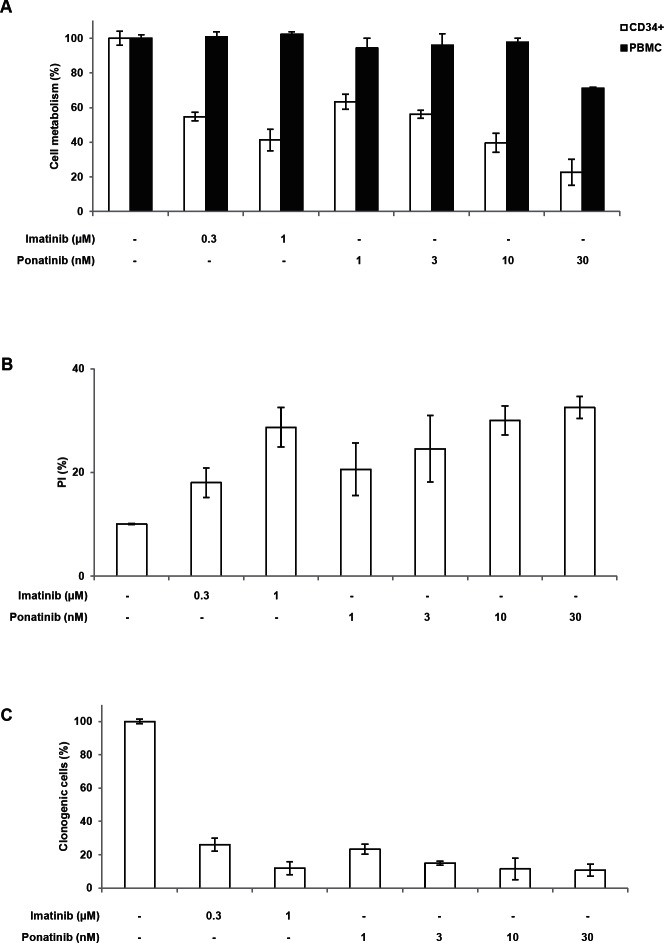
Ponatinib is highly efficient on CD34+ cells but not on PBMC from 2 CML patient at diagnosis (A and B) CD34^+^ or PBMC cells from two CML patients at diagnosis were incubated with different doses of imatinib (0.3 or 1μM) or ponatinib (1 to 30nM) for 48h in IMDM complemented with 15% BIT, 100ng/ml SCF, 100ng/ml IL6 and 10ng/ml IL3. (A) Cell metabolism was assessed using the XTT assay as described in the materials and methods section. (B) Cells were stained with the PI staining kit according to the manufacturer's indications. (C) CML cells (10'10^3^) growing in semi-solid methylcellulose medium were incubated with different concentrations of imatinib (0.3 or 1μM) or ponatinib (1 to 30nM). Results are expressed as the percentage of colony forming cells after drug treatment in comparison with the untreated control cells.

Finally, we assessed the effect of both imatinib and ponatinib on CD34+ cells from two CML patients at diagnosis. As expected Imatinib decreased CD34+ cell viability but failed to affect the viability of PBMC from the same patients. Ponatinib (30nM) was highly effective on CD34+ cells from the same patients with only a minor effect on PBMC at 30nM (Figure 4A). The effect of both TKIs accounted for by increased apoptosis as judged by AnnexinV/PI staining (Figure 4B). Finally both TKIs exhibits potent anti-proliferative effects on CD34+ cells as attested by a drastic loss of the ability of CD34+ to form colonies in soft agar (Figure 4C).

## DISCUSSION

The findings described in the present study confirm and extend the work previously reported by O’ Hare et al. in sensitive CML cell lines and BaF3 cells carrying native and mutated BCR-ABL [12]. However, our work is the first to investigate the effect of three different TKIs on a panel of imatinib-resistant cell lines. Our results indicate that ponatinib is effective on all CML cell lines in vitro, whatever the mode of resistance to imatinib. Interestingly, in our hands the cells carrying the G250E mutation seems less sensitive to the effect of ponatinib that the one carrying the T315I mutation. Indeed, dephosphorylation of BCR-ABL and CRKL by ponatinib is less effective in BaF3 carrying the G250E mutation. This is consistent with recent data from the literature that show an uncoupling between the IC50 values for BaF3 cellular proliferation assays and CRKL dephosphorylation by ponatinib [12]. Therefore, one interesting observation of the present study is the partial disconnection between BCR-ABL and CRKL phosphorylation.

CRKL is a direct substrate of BCR-ABL and therefore the status of CRKL phosphorylation is sought to be a reflect of the one of BCR-ABL. Although dephosphorylation of BCR-ABL by ponatinib was nearly complete at 10-30nM, only a higher concentration of ponatinib (100nM) was capable to efficiently dephosphorylate CRKL. These results are in good agreement with those of O Hare et al. [12]. One possible explanation for the partial disconnection of BCR-ABL and CRKL phosphorylation would be that in imatinib-resistant cells, reactivation of other kinases including ERK1/2, Fyn and/or Lyn [8, 10] contributes to the regulation of CRKL phosphorylation independently on BCR-ABL. As higher concentrations of ponatinib are required to inhibit SRC kinases, this would bring a good explanation for the lesser sensitivity of Imatinib-resistant cells to ponatinib. In line with this hypothesis, we have previously reported that a Lyn/ERK1/2 axis is required for imatinib-resistance in K562 cells [9]. Therefore, it is tempting to speculate that the Lyn/ERK1/2 module could regulate CRKL phosphorylation independently of BCR-ABL. This hypothesis is reinforced by the observation that Lyn has been shown to recruit CRKL in neutrophils [21].

In conclusion, we show here for the first time that ponatinib is highly efficient to induce cell death in different imatinib-resistant CML cell lines. Phase II clinical trial evaluation of oral ponatinib in patients with refractory CML and other hematologic malignancies are ongoing. Our results suggest that in addition to T315I patients, ponatinib could be widely used in resistant CML patients, whatever the supposed mode of resistance to first line ITKs.

## MATERIALS AND METHODS

### Reagents and antibodies

Imatinib mesylate (STI571, Gleevec) was purchased from Enzo Life Sciences (Farmingdale, NY, USA), and Dasatinib and Ponatinib were purchased from Selleckchem (Houston, TX, USA). RPMI 1640 medium, IMDM medium and fetal calf serum (FCS) were from life technologies (Carlsbad, CA, USA). Sodium fluoride, sodium orthovanadate, phenyl-methyl-sulfonyl fluoride (PMSF), aprotinin, leupeptin were purchased Sigma-Aldrich (France). Anti-HSP60 and anti-ERK antibodies were from Santa Cruz Biotechnology (Santa Cruz, CA, USA). HRP conjugated anti-mouse and anti-goat antibodies were from Dakopatts (Glostrup, Denmark). Anti-PARP, anti-phospho-ERK, anti-CRKL, anti-phospho-CRKL, anti-ABL, anti-phospho-ABL and peroxydase-conjugated anti-rabbit antibodies were obtained from Cell Signaling Technology (Beverly, MA, USA).

### Cell lines

Human CML cell lines K562, Lama, and JURLMK-1 were grown at 37°C under 5% CO2 in RPMI supplemented with 10% FCS, 50 U/ml penicillin, 50 μg/ml streptomycin, and 1mM sodium pyruvate. K562, Lama and JURLMK-1 cells imatinib-resistant have been described earlier [8, 11]. The BaF3 p210 BCR ABL WT, T31I and G250E cells were kindly provided by Pr. FX Mahon and have been described previously[22].

### Cell viability

Cells (15'103 cells/100ml) were incubated in a 96 well plate with different effectors for the times indicated in the figure legends. Fifty microliters of sodium 3-[1-phenylaminocarbonyl)-3,4-tetrazolium]-bis(4-methoxy-6-nitro) benzene sulfonic acid hydrate (XTT) reagent was added to each well. The assay is based on the cleavage of the yellow tetrazolium salt XTT to form an orange formazan dye by metabolically active cells. The absorbance of the formazan product, reflecting cell viability, was measured at 490 nm. Each assay was performed in quadruplicate.

### Flow cytometry

After stimulation, cells were washed with ice-cold PBS and were stained with the annexin-V-fluos staining kit (Roche, Meylan, France) according to the manufacturer's procedure. Fluorescence was measured by using the FL2 channels of a fluorescence-activated cell sorter apparatus (Miltenyi cytometer).

### Western blot

After stimulation, cells were harvested and lysed in buffer containing 1% Triton X-100 and supplemented with protease and phosphatase inhibitors (Roche Diagnostics). Lysates were pelleted, and 50μg of protein were analyzed by SDS-PAGE as described previously [23].

### Colony formation assay

TKI was added to cell lines growing in semisolid methylcellulose medium (0.5'103 cells/ml; MethoCult H4236; StemCell Technologies Inc, Vancouver, BC, Canada). Colonies were detected after 10 days of culture by adding 1 mg/ml of 3-(4,5-dimethylthiazol- 2-yl)-2,5-diphenyltetrazolium bromide (MTT) reagent and were scored by Image J quantification software (U.S. National Institutes of Health, Bethesda, MD, USA).

### Primary cell isolation

Blood samples were collected from patients newly diagnosed with CML. All patients were part of an institutional protocol. Peripheral blood mononuclear cells were isolated by density centrifugation (Ficoll-Paque Plus, Life Sciences). CD34+ cells were prepared by magnetic bead separation as described previously [20]. CD34+ cells from CML patients were grown at 37°C under 5% CO2 in IMDM (Gibco BRL) supplemented with 15% BIT (BSA Insulin Transferin) (Stem Cell Technologies), 100 ng/ml SCF, 100 ng/ml IL6 and 10 ng/ml IL3 were purchased from Miltenyi Biotec.

## Supplementary Figures


