# Supplementary material for: All tyrosine kinase inhibitor-resistant chronic myelogenous cells are highly sensitive to Ponatinib
Source: Oncotarget. 2012 Nov 14;3(12):1557–65. doi: 10.18632/oncotarget.692 (PMC3681494; doi:10.18632/oncotarget.692)
Supplement: Supplementary file 1 [file oncotarget-03-1557-s001.pdf]

All tyrosine kinase inhibitor-resistant chronic myelogenous cells are highly sensitive to Ponatinib - Cassuto et al

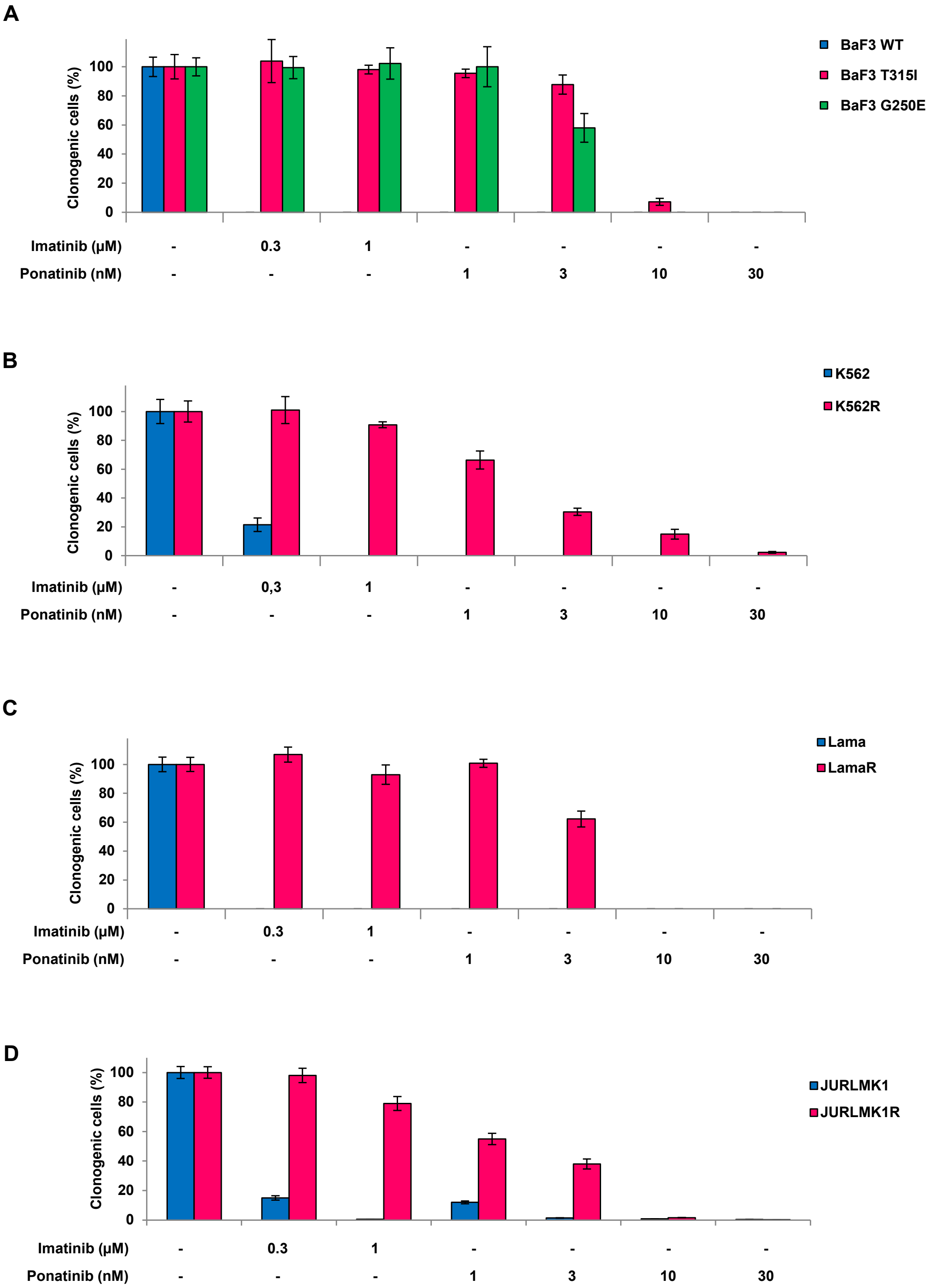

Supplemental Figure 1: Ponatinib inhibits the clonogenic potential of different TKI-resistant cell lines. (A-D) Different concentrations of imatinib (0.3 to 1µM) or ponatinib (1 to 30nM) were added to the BaF3 cell line (A) or the K562 (B), JURLMK1 (C) and Lama (D) cell lines growing in semi-solid methylcellulose medium. Results are expressed as the percentage of colony forming cells after drug treatment in comparison with the untreated control cells.
